# A novel staging system and clinical predictive nomogram for more accurate staging and prognosis of malignant pancreatic intraductal papillary mucinous neoplasms: a population-based study

**DOI:** 10.1186/s12967-021-03188-4

**Published:** 2021-12-24

**Authors:** Haoxiang Zhang, Chenggang Gao, Jiaoshun Chen, Shihong Wu, Jianwei Bai, Tao Yin

**Affiliations:** Department of Pancreatic Surgery, Union Hospital, Tongji Medical College, Huazhong University of Science and Technology, Wuhan, 430022 China

**Keywords:** American Joint Committee on Cancer, Clinical predictive nomogram, Pancreatic intraductal neoplasms, Survival

## Abstract

**Background:**

The current guidelines of the American Joint Committee on Cancer (AJCC) for the staging of exocrine pancreatic tumors seem inapplicable to malignant pancreatic intraductal papillary mucinous neoplasms (IPMN). Therefore, we aimed to improve the accuracy of clinical staging and prognosis for malignant IPMN by modifiing current AJCC system.

**Methods:**

We extracted data of 2001 patients with malignant IPMN from the Surveillance, Epidemiology, and End Results database between 2000 and 2016. Of these, 1401 patients were assigned to the primary cohort and 600 patients to the validation cohort.

**Results:**

In Kaplan–Meier analysis of the primary cohort, the current AJCC guidelines were unable to distinguish between certain tumor substages (IA and IB in the 7th, IB and IIA in the 8th). The modified system that we regrouped based on the median overall survival and hazard ratios, was superior in tumor stage classifications. Age > 70 years, tumors located in the body or tail, high-grade differentiated tumors, surgery, chemotherapy, and tumor, lymph node, and metastasis (TNM) stage were identified as independent predictive factors for overall survival. Compared to that of TNM-based systems, the concordance index of the clinical predictive nomogram significantly improved (0.819; 95% confidence interval, 0.805–0.833), with excellent area under the receiver operating characteristic curves (1-, 3-, and 5-year: 0.881, 0.889, and 0.879, respectively). The calibration curves also showed good agreement between prediction and actual observation. The analysis of treatment modalities revealed that surgery resulted in better survival for all resectable malignant IPMN. The analysis of chemotherapy data reveals its potential in improving the prognosis of treatment for patients with locally advanced or distant metastases.

**Conclusions:**

Our modified staging system improves the distinction of tumor stages. The nomogram was a more accurate and clinically reliable tool for prognosis prediction of patients with malignant IPMN.

## Background

Production of abnormally viscous mucus is a characteristic of pancreatic intraductal papillary mucinous neoplasms (IPMN). Since their first description in 1987, these rare tumors have been increasingly recognized [1]. The prevalence of IPMN is about 26 per 100,000 people; however, they are more common in the elderly, with an incidence of 99 per 100,000 people in those over the age of 60 [2, 3]. IPMN are premalignant lesions that may progress to pancreatic ductal adenocarcinoma (PDAC), and this may take several years [4]. IPMN require either surveillance or surgical resection. As stated in the histological criteria of the World Health Organization, IPMN can be classified into benign and malignant tumors. Malignant tumors can be further subdivided into high-grade dysplasia (HGD) and invasive IPMN [5, 6]. Compared to HGD (carcinoma in situ), invasive IPMN has a worse prognosis [7]. An accurate and comprehensive prognosis evaluation is particularly important. According to the 2018 European evidence-based guidelines, patients with main ductal IPMN should undergo resection [8]. All IPMN patients with jaundice, positive cytology findings, a solid component or an enhancing mural nodule measuring over 5 mm, or a main pancreatic duct measuring over 10 mm in diameter have a high risk of malignancy, and surgical excision is recommended. Surgery remains the only potentially curative treatment for malignant IPMN, and there is scope for early detection and surgical cure [6, 8].

As the most acknowledged assessment staging system for tumors, the updated American Joint Committee on Cancer (AJCC), 8th edition staging system (AJCC 8th) for exocrine pancreatic tumors has been applied clinically since 2018 [9]. Its distinction from the AJCC 7th edition staging system (AJCC 7th) lies mainly in two aspects [10]. First, because of the difficulties in determining extrapancreatic extension clinically, the definitions of T2 (> 2 cm and ≤ 4 cm in the widest diameter) and T3 (> 4 cm in the widest diameter) are now based on the criteria for invasive tumors. Second, category N is subdivided into N0 (0 regional lymph nodes are positive), N1 (one to three regional lymph nodes are positive), and N2 (four or more regional lymph nodes are positive). Minor change includes the subcategorization of T1 into T1a, T1b, and T1c based on size. Additionally, resectability was removed from the definition of T4 (Table 1). However, it has been stated that AJCC 8th is not applicable to the resection of PDAC, which accounts for 90% of pancreatic cancers [11].

**Table 1:** Definitions of AJCC TNM System

TNM 7th	TNM 8th
Tis carcinoma in situ	Tis carcinoma in situ
T1 tumor limited to the pancreas, 2 cm or less in greatest dimension	T1 tumor ≤ 2 cm in greatest dimension
T2 tumor limited to the pancreas, more than 2 cm in greatest dimension	T2 tumor > 2 cm and ≤ 4 cm in greatest dimension
T3 tumor extends beyond the pancreas but without involvement of the celiac axis or the superior mesenteric artery	T3 tumor > 4 cm in greatest dimension
T4 tumor involves the celiac axis or the superior mesenteric artery (unresectable primary tumor)	T4 tumor involves celiac axis, superior mesenteric artery, and/or common hepatic artery, regardless of size
N0 to regional lymph node metastasis	N0 to regional lymph node metastases
N1 regional lymph node metastasis	N1 metastasis in one to three regional lymph nodes
	N2 metastasis in four or more regional lymph nodes
M0 no distant metastasis	M0 mo distant metastasis
M1 distant metastasis	M1 distant metastasis

AJCC 7th	AJCC 8th	AJCC modified
Stage 0 Tis N0 M0	Stage 0 Tis N0 M0	Stage 0 Tis N0 M0
Stage IA T1 N0 M0	Stage IA T1 N0 M0	Stage IA T1 N0 M0
Stage IB T2 N0 M0	Stage IB T2 N0 M0	Stage IB T2–3 N0 M0
Stage IIA T3 N0 M0	Stage IIA T3 N0 M0	Stage IIA T1–3 N1 M0
Stage IIB T1-3 N1 M0	Stage IIB T1-3 N1 M0	Stage IIB T1-3 N2 M0
Stage III T4 N_any_ M0	Stage III T_any_ N2 M0, T4 N_any_ M0	Stage III T4 N_any_ M0
Stage IV T_any_ N_any_ M1	Stage IV T_any_ N_any_ M1	Stage IV T_any_ N_any_ M1

Our study included patients with HGD and invasive IPMN for a more comprehensive overview of malignant IPMN. We aimed to improve the predictive accuracy of current staging systems using the Surveillance, Epidemiology, and End Results (SEER) database. We modified a novel AJCC-based system to improve the distinction of tumor stages and examined an extensive series of patients with malignant IPMN to investigate predictive factors, and develop a nomogram for a more precise prediction of the prognosis of malignant IPMN.

## Methods

### Patients and data collection

**Fig. 1:**
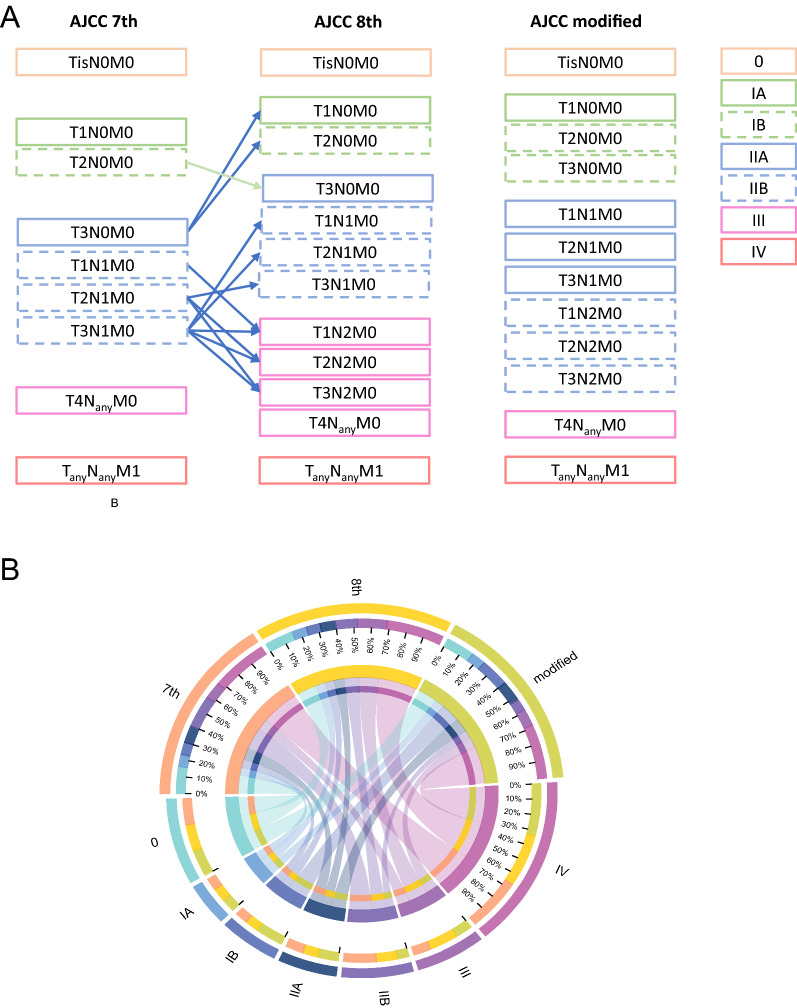
Stage systems changed between the 7th, 8th and modified edition of the AJCC staging systems (**A**); Circos plot of the distribution difference between AJCC 7th, AJCC 8th and modified stage system in this study (**B**)

This retrospective data analysis of a cohort of patients, pathologically diagnosed with malignant IPMN from the SEER database (https://seer.cancer.gov/data-software/) between 2000 and 2016, was performed using the SEER* Stats software, version 8.3.6.1 (National Cancer Institute, Rockville, MD, US). Cases were selected based on their histology, which was identified using histology codes (8050, 8260, 8450, 8453, 8471, 8480, 8481, and 8503) and ICD-O-3 topography codes (C25.0–C25.9) [12]. The tumor stages (AJCC 7th and AJCC 8th) were derived using data on tumor size and invasion, lymph node involvement, and metastasis, all of which were available in the SEER database. Data on therapy including surgery, chemotherapy, and radiotherapy were also collected and analyzed. Patients who met the following criteria were included: (1) histology or puncture cytology positive for malignant IPMN (including HGD and invasive IPMN), (2) sufficient information to allow restaging according to current AJCC guidelines (7th and 8th), and (3) age > 20 years and complete clinical and follow-up data. Patients in whom the above criteria were missing, were excluded. The definitions of and differences between AJCC 7th and AJCC 8th are shown in Table 1 and Fig. 1.

All patient data included no identifiable patient information and were accessed from the SEER database with permission. The study design was approved by the ethics committee of Union Hospital, Tongji Medical College, Huazhong University of Science and Technology, and the need for informed consent was waived owing to the study being a population study deemed not to constitute human subject research.

### Statistical analysis

The study population was divided into a primary cohort and a validation cohort at a ratio of 7:3, using the caret package of R, version 4.0.3 (http://www.r-project.org/). Survival was calculated from the date of final diagnosis until the last follow-up or death and was analyzed using Kaplan–Meier curves. Cox proportional hazards regression was used for univariate and multivariate analyses. All predictors shown to be significant in the univariate analysis were investigated using multivariate analysis. Hazard ratios (HRs) and 95% confidence intervals (CI) were analyzed. Significance was determined using log-rank tests. The concordance index (C-index) and survival curves with pairwise comparison results by the log-rank test was used to evaluate the discriminatory powers of the two different staging systems. A nomogram was constructed using the rms package within R, which included all significant independent factors in the multivariate analysis for predicting 1-, 3-, and 5-year overall survival (OS). The nomogram performance was assessed using the receiver operating characteristic (ROC) curves, C-index, and calibration curves. During the validation, the total points were calculated according to the established nomogram. Consecutively, Cox regression was performed. ROC curves, calibration curves, and C-index were derived based on the regression analysis [13].

All statistical tests were performed using the statistical language R, version 4.0.3. All tests were two-sided, and a p-value of < 0.05 was considered statistically significant.

## Results

### Baseline characteristics

A total of 2001 patients with malignant IPMN from 2000 to 2006 were enrolled in this study, of which 1401 patients were included in the primary cohort, and 600 patients constituted the validation cohort. The baseline characteristics of patients in both cohorts are shown in Table 2. In the primary cohort, the median age of patients at diagnosis was 67 years. The male-to-female ratio was similar between the cohorts. The median OS of patients was 18 months (1-year survival rate, 58.4%; 3-year survival rate, 35.9%; 5-year survival rate, 29.3%).

**Table 2:** Baseline characteristics

	The primary cohort (n = 1401)		The validation cohort (n = 600)			The primary cohort (n = 1401)		The validation cohort(n = 600)	
Age, n, %					Chemotherapy, n, %				
< 70	800	57.10%	366	61.0%	Yes	646	46.11%	300	50.0%
≥ 70	601	42.90%	234	39.0%	No/unknown	755	53.89%	300	50.0%
Sex, n, %					Radiotherapy, n, %				
Female	671	47.89%	288	48.0%	Yes	282	20.12%	120	20.0%
Male	730	52.11%	353	58.8%	No	1199	85.58%	480	80.0%
Marital status, n, %									
Married	863	61.60%	353	58.8%					
Others^a^	538	38.40%	247	41.2%					
					AJCC 7th, n, %				
Race, n, %					0	222	15.85%	98	16.3%
Others	151	10.78%	59	9.8%	IA	98	7.00%	20	3.3%
Black	1096	78.23%	475	79.2%	IB	100	7.14%	41	6.8%
White	154	10.99%	66	11.0%	IIA	141	10.06%	66	11.0%
		0.00%			IIB	260	18.56%	101	16.8%
Location, n, %					III	138	9.85%	74	12.3%
Head	762	54.39%	339	56.5%	IV	442	31.55%	200	33.3%
Body or tail	387	27.62%	160	26.7%					
Others^b^	353	25.20%	101	16.8%	AJCC 8th, n, %				
					0	222	15.85%	98	16.3%
Grade, n, %					IA	113	8.07%	33	5.5%
High^c^	191	13.63%	78	13.0%	IB	107	7.64%	48	8.0%
Others^d^	1210	86.37%	522	87.0%	IIA	119	8.49%	46	7.7%
					IIB	168	11.99%	66	11.0%
ELN, n, %					III	230	16.42%	109	18.2%
< 15	1012	72.23%	448	74.7%	IV	442	31.55%	200	33.3%
≥ 15	389	27.77%	152	25.3%					
					AJCC modified, n, %				
Extend, n, %					0	222	15.85%	98	16.3%
Inside	666	47.54%	275	45.8%	IA	113	8.07%	33	5.5%
Beyond	735	52.46%	325	54.2%	IB	226	16.13%	94	15.7%
					IIA	168	11.99%	66	11.0%
Surgery, n, %					IIB	92	6.57%	35	5.8%
Yes	862	61.53%	343	57.2%	III	138	9.85%	74	12.3%
No	539	38.47%	257	42.8%	IV	442	31.55%	200	33.3%

^a^“Others” in marital status include single (never married), separated, divorced, widowed, unmarried or domestic partner and unknown

^b^“Others” in location include pancreatic duct, others specified part of pancreas, overlapping lesion of pancreas and pancreas, NOS

^c^“High” in grade include poorly differentiated and undifferentiated

^d^“Others” in grade include well, moderately and unknown

The major difference between AJCC 7th and AJCC 8th lay in the IA, IB, IIA, IIB, and III stages. In the primary cohort, 7.00%, 7.14%, 10.06%, 18.56%, and 9.85% of patients were in stages IA, IB, IIA, IIB, and III, respectively, when using AJCC 7th. In contrast, according to AJCC 8th, 8.07%, 7.64%, 8.49%, 11.99%, and 16.42% of patients were in IA, IB, IIA, IIB, and III stages, respectively.

### Predictive prediction of current stage systems and stage modification

**Fig. 2:**
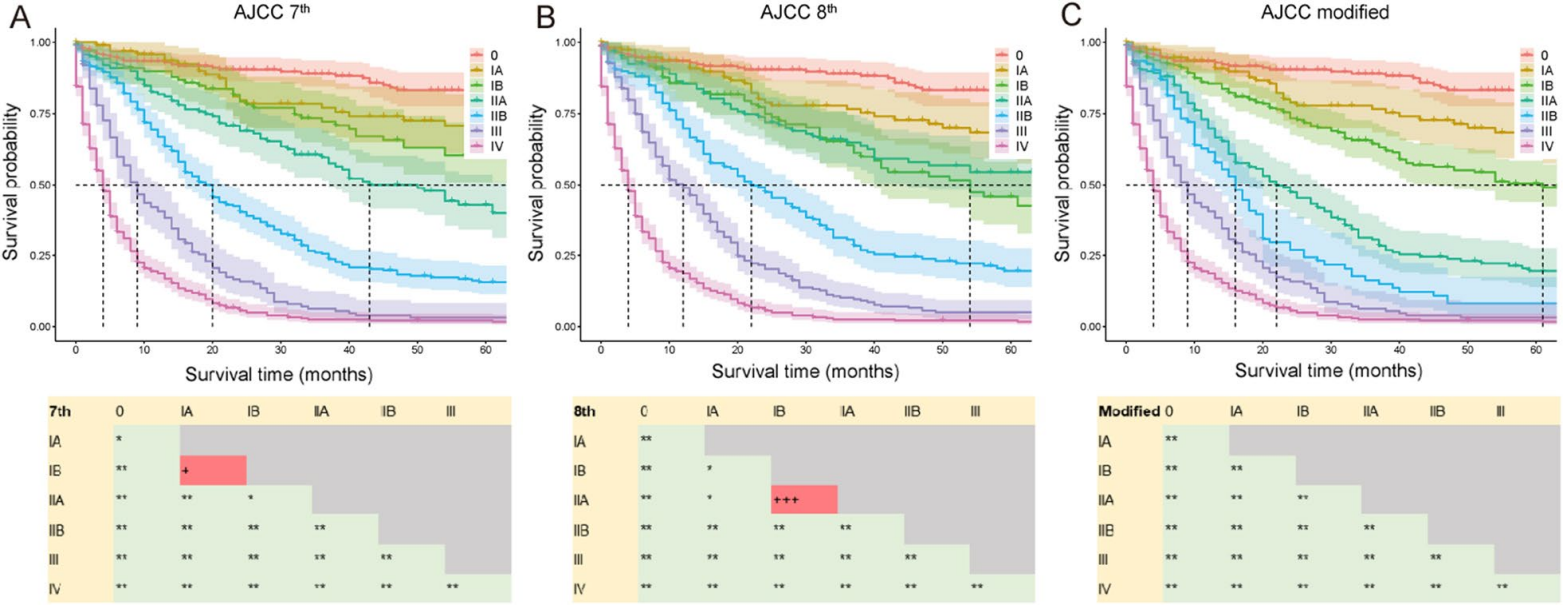
Kaplan–Meier survival curves and pairwise comparison results according AJCC 7th (**A**), AJCC 8th (**B**) and AJCC modified (**C**) in primary cohort. Significance was determined by log-rank tests. *p < 0.05; **p < 0.01; ^+^p ≥ 0.05; ^++^p ≥ 0.1; ^+++^p ≥ 0.5

In the primary cohort, the C-index using AJCC 7th and that with AJCC 8th were 0.779 (95% CI 0.755–0.803) and 0.777 (95% CI 0.753–0.801), respectively. Further pairwise comparison by the log-rank test showed that stages IA and IB when using AJCC 7th and stages IB and IIA when using AJCC 8th, were not sufficiently distinguishable (p > 0.05) (Fig. 2A, B). Similar results are shown in the Kaplan–Meier curves.

**Fig. 3:**
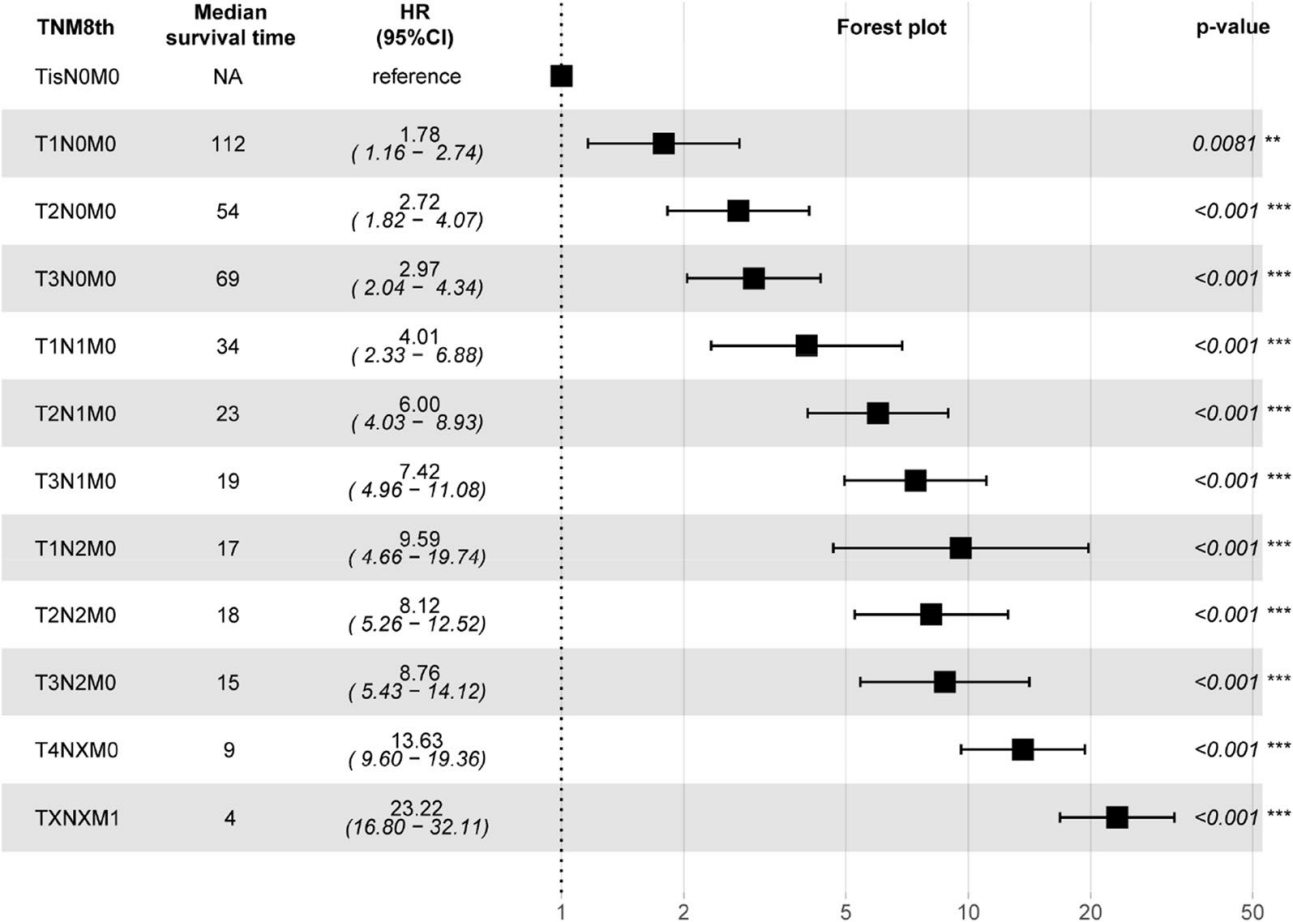
Median survival time and univariate analysis results with forest plots of AJCC 8th substages in primary cohort. *p < 0.05, **p < 0.01, ***p < 0.001

We concluded that the current AJCC 8th was not sufficiently accurate for malignant IPMN. The median OS time and univariate analysis results of patients for each substage of the AJCC 8th in the primary cohort are shown in Fig. 3. The composite measure combined these indicators, and we regrouped the substages and arrived at a modified staging system (AJCC modified) based on the median OS, pairwise comparison results, and HRs of each substage (Table 1). Although the C-index (0.779; 95% CI 0.755–0.803) did not change significantly (Table 3), the pairwise comparisons of AJCC modified were all statistically significantly different (p < 0.05). The resulting survival curves of AJCC modified are shown for the different stages in Fig. 2C.

**Table 3:** Concordance indexes of different staging systems for malignant IPMN

Stage system	The primary cohort (n = 1401)	The validation cohort (n = 600)
AJCC 7th	0.779 (0.755–0.803)	0.759 (0.735–0.783)
AJCC 8th	0.777 (0.753–0.801)	0.753 (0.729–0.777)
AJCC modified	0.779 (0.755–0.803)	0.756 (0.732–0.78)
Prognostic nomogram	0.801 (0.787–0.815)	0.791 (0.769–0.813)

In addition, the C-index of both AJCC (7th or 8th) and modified staging systems increased with time (Table 4).

**Table 4:** Concordance indexes of different staging systems in different periods for malignant IPMN

Stage system	2000–2004 (n = 345)	2005–2007 (n = 370)	2008–2010 (n = 408)	2011–2013 (n = 421)	2014–2016 (n = 457)
AJCC 7th	0.688 (0.673–0.703)	0.773 (0.761–0.785)	0.763 (0.749–0.777)	0.78 (0.766–0.794)	0.807 (0.791–0.823)
AJCC 8th	0.707 (0.691–0.723)	0.769 (0.757–0.781)	0.761 (0.747–0.775)	0.778 (0.764–0.792)	0.806 (0.790–0.822)
AJCC modified	0.69 (0.675–0.705)	0.774 (0.762–0.786)	0.765 (0.751–0.779)	0.78 (0.766–0.794)	0.807 (0.791–0.823)

### Independent predictive factors for malignant IPMN

The results of the univariate and multivariate analyses are listed in Table 5. Multivariate analyses demonstrated that age > 70 years, tumors located in the body and tail, high-grade differentiated tumors, surgery, chemotherapy, and tumor, lymph node, and metastasis (TNM) stages based on AJCC 8th were independent risk factors for OS (p < 0.05).

**Table 5:** Univariate and multivariate analysis of factors associated with OS in the primary cohort

Variable	Variable levels	Univariate analysis			Multivariate analysis		
		HR	95% CI	p-value	HR	95% CI	p-value
Age	< 70	Reference			Reference		
	≥ 70	1.37	1.21–1.56	< 0.001***	1.49	1.30–1.70	< 0.001***
Sex	Female	Reference					
	Male	0.93	0.82–1.05	0.252			
Race	Black	Reference			Reference		
	White	0.87	0.71–1.06	0.163	1.1	0.9–1.36	0.355
	Others	0.59	0.44–0.78	< 0.001***	0.78	0.58–1.05	0.099
Marriage	Others	Reference			Reference		
	Married	0.82	0.72–0.94	0.003**	0.98	0.86–1.13	0.801
Location	Head	Reference			Reference		
	Body or tail	1.70	1.47–1.96	< 0.001***	1.22	1.04–1.43	0.014*
	Others	1.24	1.04–1.48	0.015*	1.11	0.93–1.33	0.256
Grade	Others	Reference			Reference		
	High	1.38	1.16–1.64	< 0.001***	1.59	1.32–1.91	< 0.001***
ELN	< 15	Reference			Reference		
	≥ 15	0.51	0.43–0.59	< 0.001***	0.93	0.78–1.13	0.479
Extend	Inside	Reference			Reference		
	Beyond	2.26	1.97–2.58	< 0.001***	1.14	0.97–1.35	0.122
Surgery	Yes	Reference			Reference		
	No	5.14	4.48–5.90	< 0.001***	3.6	2.88–4.49	< 0.001***
Chemotherapy	Yes	Reference			Reference		
	No/unknown	0.79	0.69–0.89	< 0.001***	2.04	1.77–2.36	< 0.001***
Radiotherapy	Yes	Reference					
	No/unknown	0.98	0.84–1.14	0.690			
T8th	Tis	Reference			Reference		
	T1a	1.4	0.74–2.66	0.301	1.68	0.88–3.2	0.115
	T1b	1.93	0.91–4.11	0.088	2.58	1.21–5.52	0.014*
	T1c	4.16	2.86–6.05	< 0.001***	3.93	2.66–5.79	< 0.001***
	T2	6.59	4.78–9.09	< 0.001***	5.13	3.62–7.27	< 0.001***
	T3	6.7	4.86–9.23	< 0.001***	5.19	3.68–7.33	< 0.001***
	T4	12.99	9.34–18.08	< 0.001***	6.16	4.18–9.07	< 0.001***
N8th	N0	Reference			Reference		
	N1	1.21	1.02–1.43	0.026*	1.55	1.28–1.88	< 0.001***
	N2	1.47	1.17–1.83	< 0.001***	2.94	2.25–3.84	< 0.001***
M8th	M0	Reference			Reference		
	M1	5.58	4.84–6.42	< 0.001***	2.35	1.94–2.87	< 0.001***

p < 0.05; **p < 0.01; ***p < 0.001

The HRs of T2 and T3 were not significantly different (in the multivariate analysis with Tis as the reference: T2, HR = 4.97; 95% CI 3.50–7.04; T3, HR = 5.05; 95% CI 3.58–7.13). The HR of a T1a tumor was not significantly different from that of a Tis (multivariate analysis with Tis as the reference: T1a, HR = 1.68; 95% CI 0.88–3.2; p = 0.114).

### Clinical predictive nomogram for OS

**Fig. 4:**
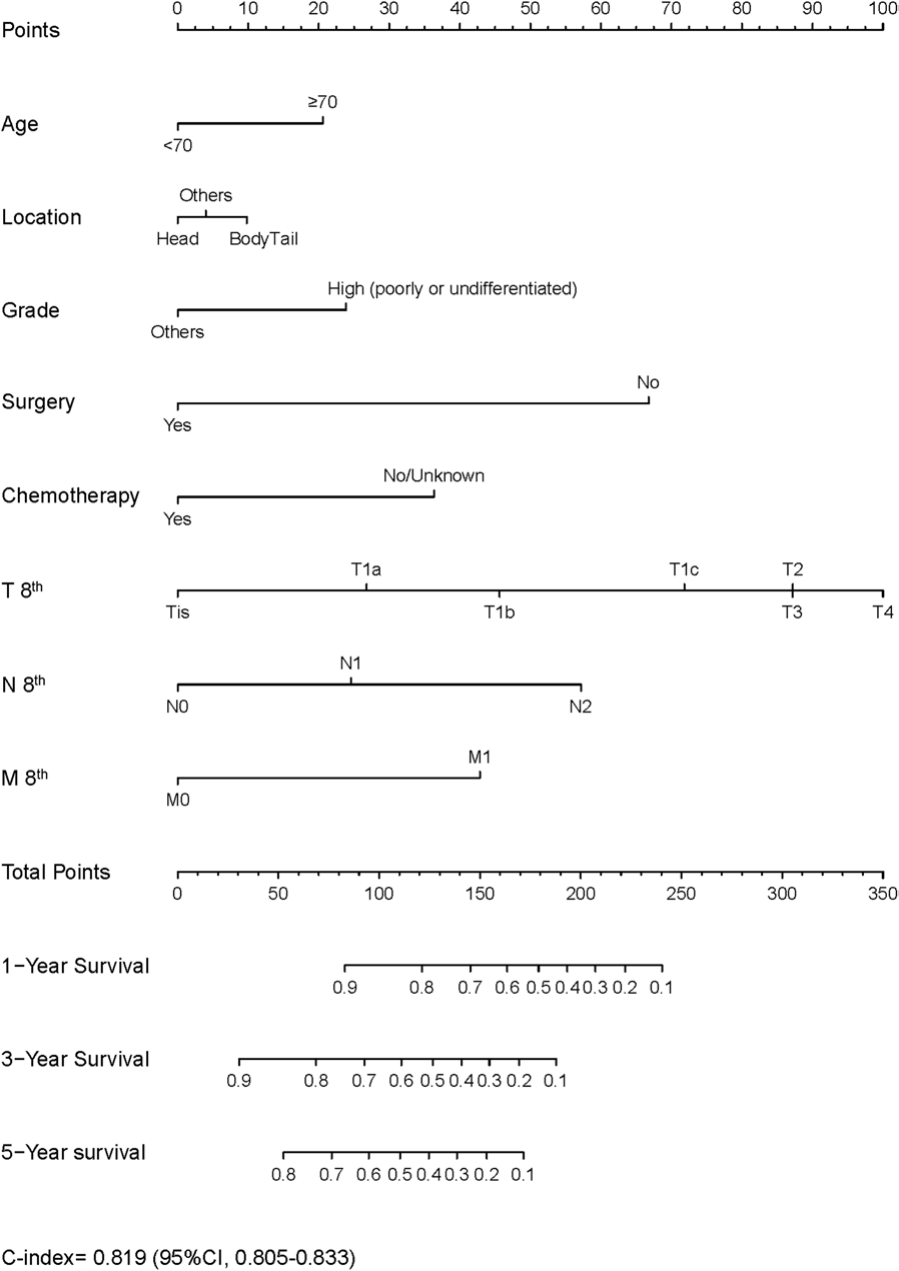
Clinical predictive nomograms for predicting 1-year, 3-year, and 5-year survival of patients with malignant IPMN

**Fig. 5:**
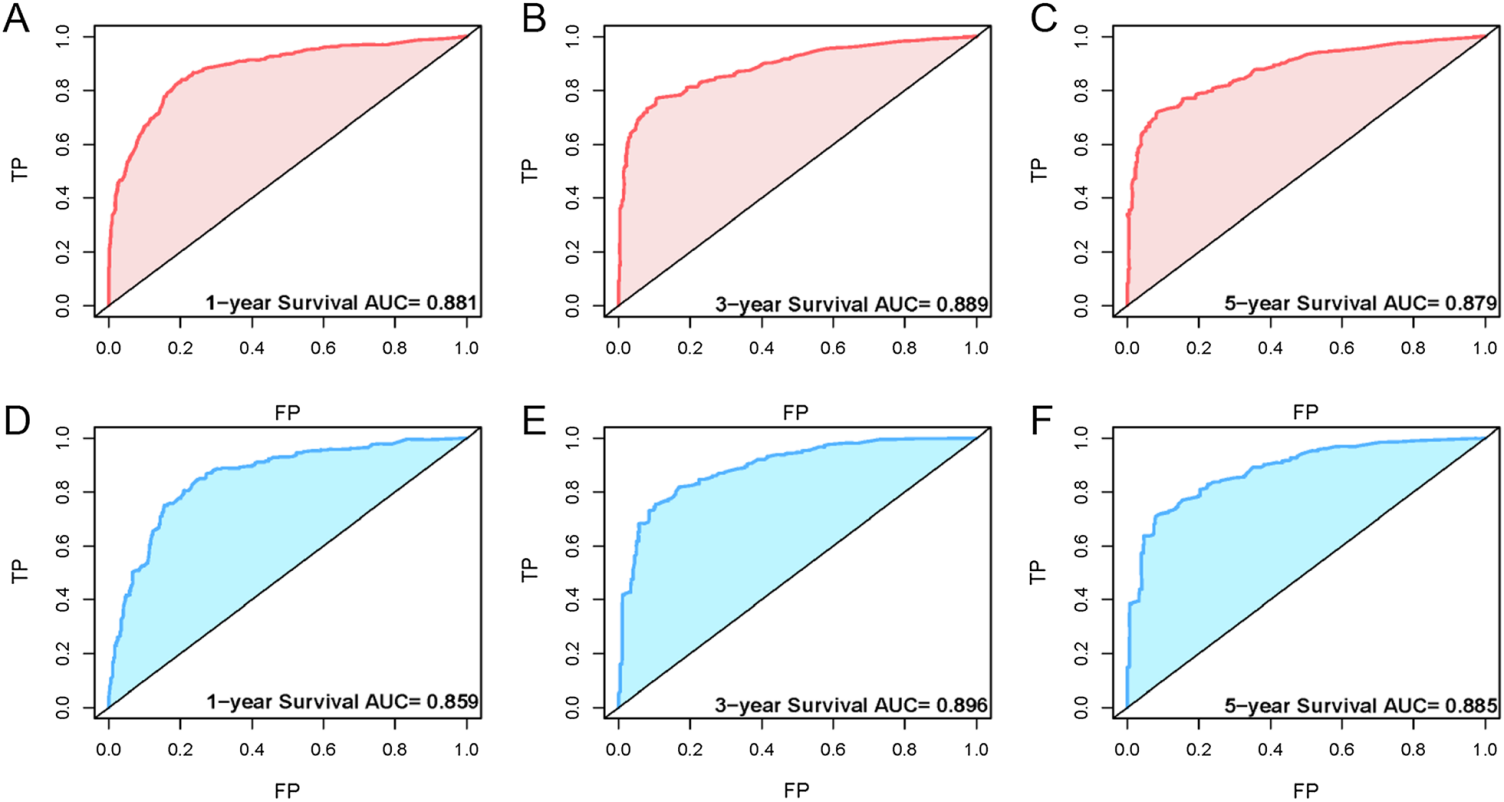
The receiver operating characteristic curve of clinical predictive nomogram for predicting patient survival at 1-year (**A**), 3-year (**B**) and 5-year (**C**) in the primary cohort. The receiver operating characteristic curve of clinical predictive nomogram for predicting patient survival at 1-year (**D**), 3-year (**E**) and 5-year (**F**) in the validation cohort (FP = false-positive; TP = true-positive)

**Fig. 6:**
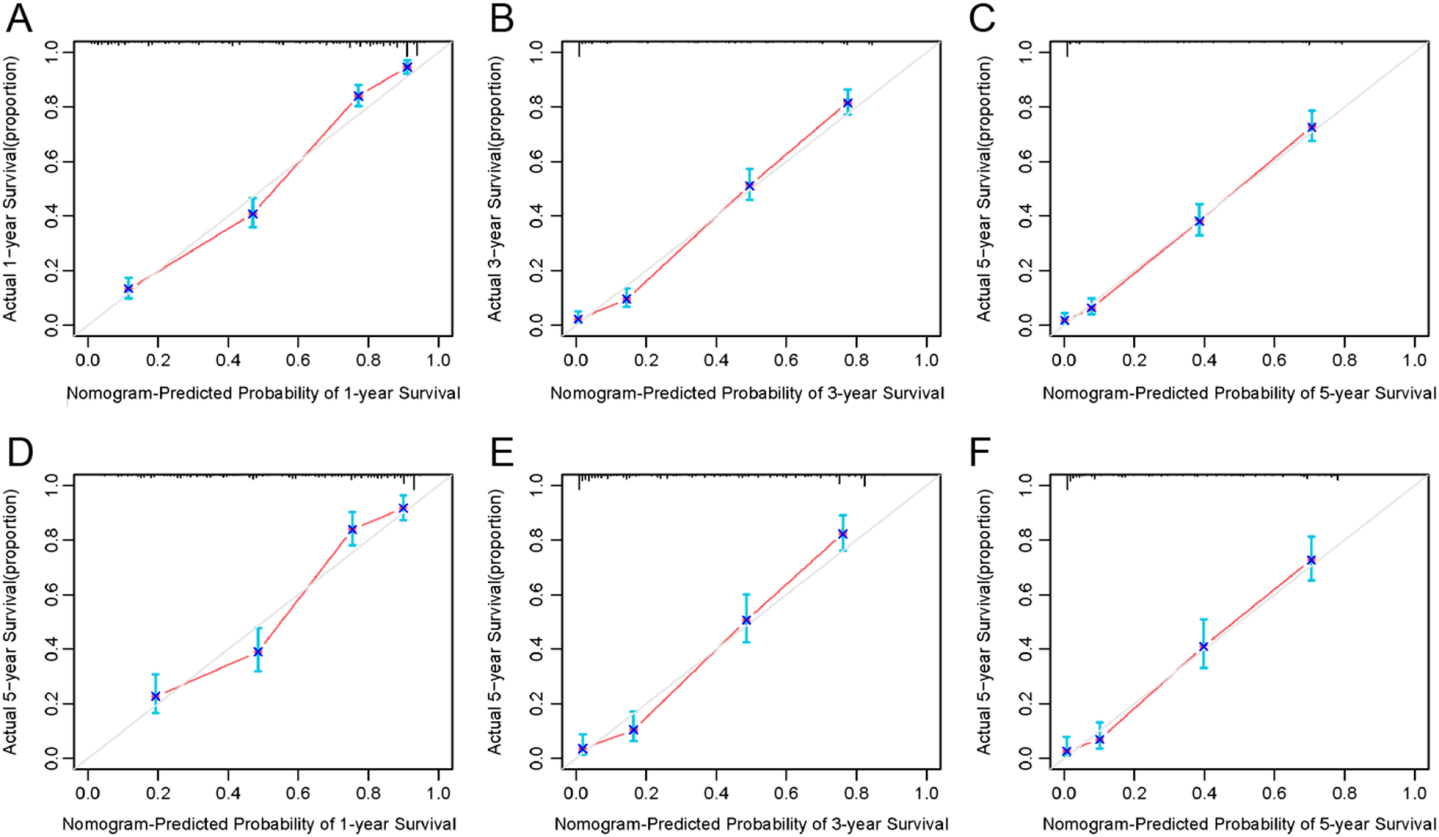
The calibration curve of clinical predictive nomogram for predicting patient survival at 1-year (**A**), 3-year (**B**) and 5-year (**C**) in the primary cohort. The calibration curve of clinical predictive nomogram for predicting patient survival at 1-year (**D**), 3-year (**E**) and 5-year (**F**) in the validation cohort

The clinical predictive nomogram was developed using the predictive determinants of OS identified in the multivariate analysis (Fig. 4). The contribution of a predictor to OS can be quantified by the length of the line corresponding to each variable in the clinical predictive nomogram. We found that the T stage of AJCC 8th made the most significant contribution to survival, closely followed by surgery, the N stage, chemotherapy, and the M stage. The nomogram showed a high predictive precision, with the C-index being 0.819 (95% CI 0.805–0.833). The 1-, 3-, 5-year calibration curves showed a significant agreement between prediction and observation in the probability of survival (Fig. 5A–C). A similar precision was shown by the ROC curves. The values of the 1-, 3-, and 5-year OS area under the curve (AUC) were 0.881, 0.889, and 0.879, respectively (Fig. 6A–C).

### Validation of the clinical predictive nomogram for OS in the validation cohort

The median age of patients at diagnosis in the validation cohort was 66 years, and the median OS of patients was 18 months (1-, 3-, 5-year survival rates: 59.4%, 36.9%, and 30.7%, respectively).

The C-index of the established nomogram in the validation cohort was 0.791 (95% CI 0.769–0.813). The 1-, 3-, and 5-year calibration curves (Fig. 5D–F) and the 1-, 3-, and 5-year AUC values (Fig. 6D–F) also presented ideal agreements within the primary cohort.

### Effect of clinical interventions on OS in the AJCC modified system

**Fig. 7:**
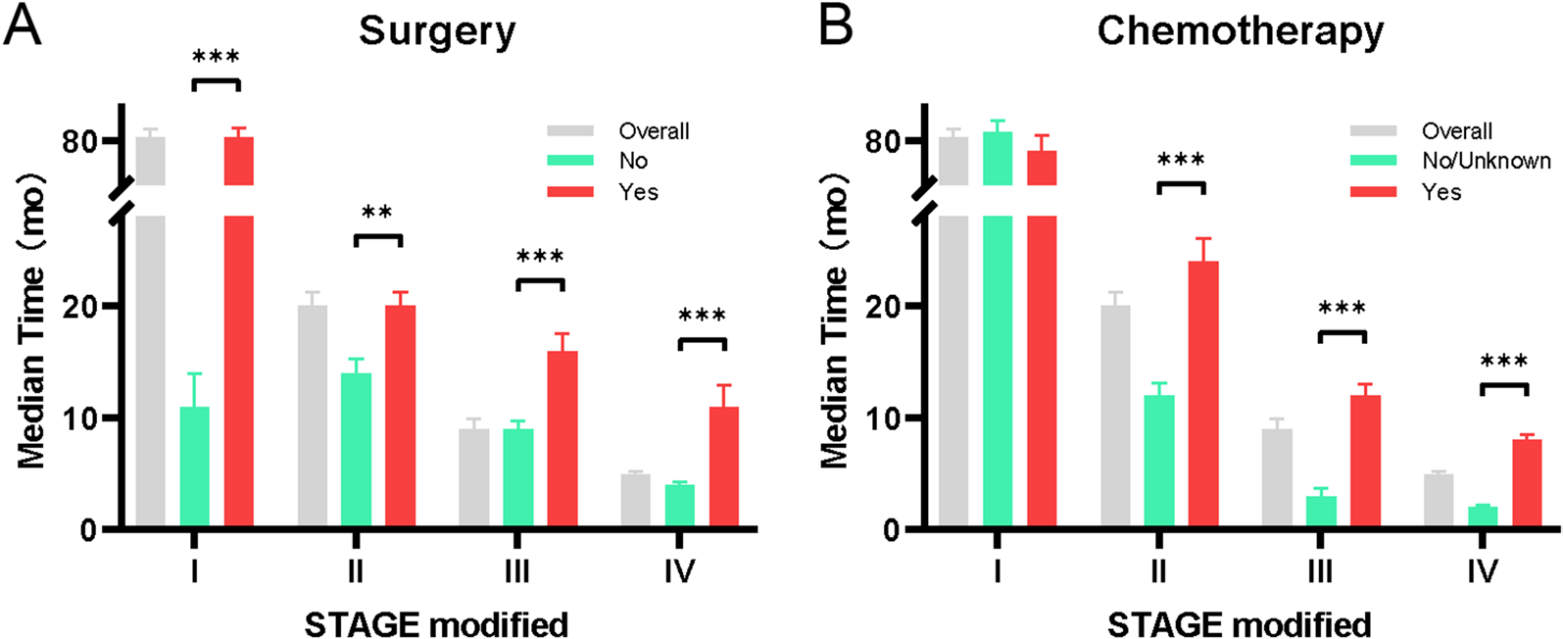
The median OS with surgery (yes or no) (**A**), chemotherapy (yes or no/unknown) (**B**) in stage I, II, III and IV based on AJCC modified in the entire cohort. Significance was determined by log-rank tests. *p < 0.05; **p < 0.01; ***p < 0.001

Surgery, chemotherapy, and radiotherapy are the main clinical interventions for malignant IPMN. In the multivariate analysis, surgery and chemotherapy were statistically significantly associated with OS (p < 0.05). To further evaluate the effect of clinical interventions in the different substages, the median OS of patients who underwent surgery and chemotherapy in each substage (I–IV) was also analyzed for the AJCC modified system. The results are presented in Fig. 7. Across all the substages (I–IV), patients who underwent surgery had a significantly longer survival time than those who did not (p < 0.05, log-rank test). Within the II, III, and IV substages, patients who received chemotherapy had a significantly longer survival time than those who did not receive chemotherapy or when their status was not known.

## Discussion

Based on the TNM system, the AJCC staging manual has become a standardized classification system for evaluating cancer at a population level in terms of the extent of disease [14]. We first evaluated the predictive value of the last two AJCC staging systems using the SEER database to assess the need for revision. The C-index is the most widely used index to assess a model’s differentiation power to correctly predicting survival. The C-indexes of the AJCC 7th and AJCC 8th in both the primary cohort (0.779 and 0.777, respectively) and the validation cohort (0.759 and 0.753, respectively) were not significantly different. The high predictive ability of the guideline analysis system and the significant prognostic differences in different stages can guide doctors in assessing the severity of the disease and selecting appropriate intervention. Our pairwise comparison by the log-rank test and Kaplan–Meier curves showed that outcomes of stage IA in AJCC 7th and stage IIA in AJCC 8th are not significantly different compared to those of stage IB. This finding indicates that the modifications from AJCC 7th to 8th did not significantly alter its clinical applicability and predictive differentiation ability, and that there are no significant differences among some of the stages in both AJCC 7th and 8th staging systems. Both systems should be further improved for malignant IPMN. Therefore, we compared the median survival time and univariate analysis results of patients in each substage of AJCC 8th and proposed a modified staging system. The modified staging system distinguished all substages sufficiently (p < 0.05).

Furthermore, we found that the C-indexes were increasing with time in the same evaluation system; this could be closely related to the development of medical imaging techniques [15].

Nomograms have been shown as more accurate tools than the conventional staging systems for predicting prognosis in many cancers [16–18]. Age, tumor location, differentiation grade, surgery, chemotherapy, and TNM stage in the AJCC 8th were independent factors for survival in multivariate analysis (p < 0.05), therefore, we developed the clinical predictive nomogram. The C-index was 0.819, which was statistically higher than that of the TNM-based stage systems (AJCC 7th, AJCC 8th and AJCC modified) in this study. Furthermore, the 1-, 3-, and 5-AUCs of the nomogram were close to 0.90, supporting its ability to predict individual survival accurately to a certain degree. However, our clinical predictive nomogram is more than a tool to predict survival. Furthermore, the length of the line corresponding to each variable quantifies its contribution to predicting survival.

Tumor stage was the most important predictor of malignancy in malignant IPMN with the longest line in the nomogram. T-stages are mainly based on tumor size. First, we found that the HR of T1a is not significantly different from that of Tis (p < 0.05). Invasive IPMN with tumor size < 0.5 cm (T1a) can be characterized as minimally invasive, which has roughly the same outcome as HGD IPMN. However, we noted that only a tumor size < 2 cm was an independent predictive factor; the length of the line in the nomogram and the HR of T2 and T3 were the same. The distinction of a T3 seems to be of limited predictive value, which may be the key factor affecting the accuracy of AJCC 8th for IPMN. This manifests the “degeneracy” of TMN scoring, in the condition as well as in general application in cancer, i.e. that multiple TMN scores are associated with the same stage.

As shown in previous studies, positive lymph nodes play a key role in the prognosis of IPMN [19, 20]. The prognoses of patients with N2 tumors are significantly different from those of patients with N1 and N0 tumors, which shows that the number of positive lymph nodes is one of the independent predictive factors for malignant IPMN in our study. Significant differences can also be seen in the median survival time of patients with different N stages. An adequate number of examined lymph nodes (ELNs) is necessary to evaluate N staging. The more local lymph nodes are examined, the more accurate the N staging becomes. Regional lymph node metastases are frequent in patients with invasive IPMN (26.3%, 447/1699 in the entire cohort). This finding is consistent with those of previous reports [4, 21]. Based on the above studies, lymph node dissection similar to that done for PDAC might be necessary for malignant IPMN. However, in our study, multivariate analysis showed that even a number of ELNs > 15 made no significant difference in survival.

Our results support the concept that malignant IPMN located in the pancreatic head have a better OS than those in the body or tail (head vs. body or tail, HR = 1.22, p = 0.014). These results support the findings of most previous studies on IPMN [22–24]. Kerlakian et al., demonstrated that jaundice was more often seen in patients with uncinate or head cysts (14.9% vs. 1.9%, p < 0.01) and that incidentally discovered or asymptomatic IPMN were more likely in patients with tumors located in the neck, body, or tail of the pancreas (53.3% vs. 31.0%, p < 0.01) [25]. Furthermore, the median time from diagnosis to surgery was shorter. The insidious nature of symptoms in the early stages of IPMN located in the body and tail may explain why these patients have worse outcomes. Moreover, a Japanese study showed that body or tail pancreatic IPMN is one of the independent risk factors for metachronous high-risk lesions in the remanent pancreas [21].

Surgery remains the mainstay of treatment in properly selected patients with malignant IPMN. Performing surgery resulted in significantly better survival in patients with the same stage of disease as in our study, and even in patients with distant metastasis. However, outcomes after surgical resection show that once malignant IPMN progresses to invasive, or even HGD, recurrence is common [4, 7, 26].

The oncological benefits of adjuvant therapy for malignant IPMN remain controversial. Therefore, we deliberately included radiotherapy and chemotherapy as parameters in this study. Chemotherapy was an independent predictive factor of survival. The median survival time significantly improved for patients with stages II, III, and IV (AJCC modified), which suggests that chemotherapy may result in better survival in patients with locally advanced cancer or distant metastases. Some retrospective studies support this notion [19, 27]. Although the analysis of chemotherapy data reveals its potential in improving the prognosis of treatment. It is worth noting that SEER chemotherapy and surgery data are incomplete and may not generally be used for outcomes research [28]. Therefore, the benefits of adjuvant therapy needs to be confirmed through large-scale studies in the future.

A long follow-up duration and a large patient population are the strengths of our study. Nevertheless, there are limitations in this study. First, it was a long-term, large-sample retrospective study; therefore, our findings need to be confirmed in a prospective cohort. With technological improvements (including diagnostic procedures and laboratory testing), different outcomes may emerge in future research. Second, although all stages were sufficiently distinguished in the modified system, the predictive ability did not significantly increase as compared to the AJCC systems; the TNM staging alone seems inadequate to further improved accurately predict the clinical outcomes of patients with malignant IPMN. Third, erroneous data or incorrect coding are still possible in the SEER database. Despite these limitations, our study of the predictive factors in malignant IPMN provides critical information for future guidelines and prospective studies.

## Conclusions

We compared the accuracy of the survival prognosis of the current two AJCC guidelines and proposed a modified system to overcome their limitations. Our analysis of independent predictive factors in malignant IPMN enabled us to build an accurate and practical clinical predictive nomogram that showed a strong objective predictive power when validated. The limited predictive ability of T3 may be a key factor that affects the accuracy of AJCC 8th for malignant IPMN. Surgery remains the only potentially curative treatment and could help improve the poor prognosis of all malignant IPMN patients. For patients with locally advanced tumors or distant metastases, chemotherapy may result in better survival. Owing to the retrospective nature of our study, further prospective studies are required.

## Data Availability

The datasets used and/or analyzed during the current study are available from the corresponding author on reasonable request.

## Abbreviations

AJCC: American Joint Committee on Cancer
IPMN: Intraductal papillary mucinous neoplasms
PDAC: Pancreatic ductal adenocarcinoma
HGD: High-grade dysplasia
AJCC 8th: AJCC 8th edition staging system
AJCC 7th: AJCC 7th edition staging system
SEER: Surveillance, Epidemiology, and End Results
HRs: Hazard ratios
CI: Confidence intervals
C-index: Concordance index
OS: Overall survival
ROC: Receiver operating characteristic
ELNs: Examined lymph nodes
